# Fluorine-18 labeled amino acids for tumor PET/CT imaging

**DOI:** 10.18632/oncotarget.19943

**Published:** 2017-08-04

**Authors:** Yiqiang Qi, Xiaohui Liu, Jun Li, Huiqian Yao, Shuanghu Yuan

**Affiliations:** ^1^ School of Medicine and Life Sciences, University of Jinan-Shandong Academy of Medical Sciences, Jinan, Shandong, China; ^2^ Department of Radiation Oncology, Shandong Cancer Hospital and Institute, Shandong Cancer Hospital affiliated to Shandong University, Jinan, Shandong, China; ^3^ Shandong Academy of Medical Sciences, Jinan, Shandong, China; ^4^ Department of Thoracic Surgery, Shandong Provincial Hospital, Jinan, Shandong, China; ^5^ Department of Surgery, Juye Coalfield Central Hospital, Heze, Shandong, China

**Keywords:** Fluorine-18 labeled amino acids, positron emission tomography/computed tomography (PET/CT), tumor metabolism, molecular imaging

## Abstract

Tumor glucose metabolism and amino acid metabolism are usually enhanced, ^18^F-FDG for tumor glucose metabolism PET imaging has been clinically well known, but tumor amino acid metabolism PET imaging is not clinically familiar. Radiolabeled amino acids (AAs) are an important class of PET/CT tracers that target the upregulated amino acid transporters to show elevated amino acid metabolism in tumor cells. Radiolabeled amino acids were observed to have high uptake in tumor cells but low in normal tissues and inflammatory tissues. The radionuclides used in labeling amino acids include ^15^O, ^13^N, ^11^C, ^123^I, ^18^F and ^68^Ga, among which the most commonly used is ^18^F [1]. Available data support the use of certain ^18^F-labeled AAs for PET/CT imaging of gliomas, neuroendocrine tumors, prostate cancer and breast cancer [2, 3]. With the progress of the method of ^18^F labeling AAs [4–6], ^18^F-labeled AAs are well established for tumor PET/CT imaging. This review focuses on the current status of key clinical applications of 18F-labeled AAs in tumor PET/CT imaging.

## INTRODUCTION

The clinical applications of tumor PET imaging are very extensive, including diagnosis, confirming status of lymph node and distant metastasis, and evaluating of curative effect. ^18^F-labeled AAs have been used for tumor PET imaging for decades, these are an important class of PET imaging agents that target the increased levels of AA transport by many types of tumor cells. System L AA transporter has been a major focus of imaging agents development, and work in this field has led to several ^18^F-labeled AAs as PET tracers, such as ^18^F-FET, ^18^F-FDOPA, ^18^F-D-FMT, ^18^F-FAMT, ^18^F-OMFD, and ^18^F-FACBC. Recently, emerging ^18^F-labeled AAs have been developed that target system A, xCT, glutamine, and cationic amino acid transporters [7]. So far, the main clinical applications of ^18^F-labeled AAs are gliomas, neuroendocrine tumors, prostate cancer and breast cancer PET/CT imaging.

### Mechanism of amino acid metabolism for tumor PET imaging

Certain AA transporters, particularly LAT1 and ASCT2 [8–10], are upregulated in a wide range of different types of tumors, there is growing evidence that some AA transporters and their substrates interact with the mammalian target of rapamycin (mTOR) pathway, which regulates cell proliferation and protein synthesis [11, 12]. These upregulated AA transporters increase much more amino acid uptake of tumors. ^18^F-labeled amino acids are an important class of tumor imaging agents suitable for PET/CT. PET is a kind of radiotracer-based imaging method, which can provide unique, noninvasive molecular and functional information about tumor biology that complements more anatomically based modalities, such as magnetic resonance imaging (MRI) and computed tomography (CT). ^18^F-labeled AAs detect increased tumor amino acid metabolism levels by targeting upregulated AA transporters in PET imaging, the key of that is the amino acid transport system [1, 2, 13, 14]. Amino acids enter cells through membrane-associated transporter, and more than 20 amino acid transporters have been discovered in mammalian cells [15–18]. According to the need for sodium ions, amino acid transport system can be divided into the following two categories [10, 19–21]: (1) Na^+^-dependent amino acid transport systems, including system ASC (alanine-serine-cysteine preferred), system A (alanine preferred), system N (glutamine, aspartic acid and histidine preferred), X- AG(transport L-glutamic acid, D-/L-aspartic acid) and B^0+^(transport neutral and basic amino acids); (2) Na^+^-undependent amino acid transport systems, including system L (leucine preferred), y^+^ (CAT) (selectively transport basic amino acids), y^+^L (transport neutral and basic amino acids), b^0+^ (transport neutral and basic amino acids) and X- C (transport cystine and glutamic acid). The system A, system L and system ASC are the most common amino acid transport systems [16, 22–26].

### PET tracers based on ^18^F-labeled amino acids

^18^F-labeled amino acids are an class of the most commonly used tracers for tumor PET imaging, the ideal PET tracers based on ^18^F-labeled AAs should conform to the following conditions: (1) can be quickly transported to the tumor cells, and have a high uptake rate and a certain retention time; (2) do not combine with non-protein and inflammatory tissue; (3) have a high plasma clearance rate; (4) have a better blood-brain barrier permeability for the brain tumors; (5) have a relatively simple and practical labeling method [18, 27]. At present, clinical commonly used ^18^F-labeled amino acids are basically in line with the above conditions, these are listed in Table 1.

**Table 1:** Clinical applications of ^18^F-labeled amino acids

Abbreviation	Full name of tracers	Transport system	Clinical applications
^18^F-FDOPA	L-3,4-dihydroxy-6-^18^F-fluoro-phenylalanine	System L and Amino acid decarboxylase	Brain tumors and Neuroendocrine tumors
^18^F-OMFD	3-O-methyl-6-^18^F-fluoro-L-3,4-dihydroxyphenylalanine	System L	Brain tumors
^18^F-FET	O-(2-^18^F-fluoroethyl)-L-tyrosine	System L	Brain tumors
^18^F-FAMT	L-3-^18^F-fluoro-alpha-methyl tyrosine	System L	Brain tumors, Oral cavity cancer and Non-small cell lung cancer
2-FTyr	2-^18^F-fluoro-L-tyrosine	System L	Brain tumors
^18^F-FGln	4-^18^F-(2S,4R)-fluoro-glutamine	System L and ASCT2	Brain tumors and Breast cancer
^18^F-D-FMT	O-^18^F-fluoromethyl-D-tyrosine	System L	Non-small cell lung cancer
^18^F-FSPG (BAY 94-9392)	(4S)-4-(3-^18^F-fluoropropyl)-L-glutamate	System X_C_^−^	Brain tumors, Lung cancer and Liver cancer
^18^F-FASu	^18^F-5-fluoroaminosuberic acid	System X_C_^−^	Breast cancer
^18^F-FACBC	anti-1-amino-3-^18^F-fluorocyclobutane-1-carboxylic acid	System L and ASCT2	Prostate cancer and Breast cancer
^18^F-FACPC	anti-1-amino-2-^18^F-fluorocyclopentane-1-carboxylic acid	System L and ASCT2	Prostate cancer
^18^F-Cis-FPro	cis-4-^18^F-fluoro-L-proline	System A and B^0,+^	Urinary system tumors

### Clinical applications of ^18^F-labeled amino acids in tumor PET/CT imaging

#### Gliomas

Gliomas are occurring in the neuroectodermal, are also known as neuroectodermal tumors or neuroepithelial tumors. Contrast-enhanced MRI plays a critical role in glioma imaging, including diagnosis, monitoring response to therapy, staging, and assessing for recurrence, but has limited accuracy for distinguishing between recurrence and radiation necrosis, and evaluating the nonenhancing portions of gliomas. The value of ^18^F-labeled AAs PET in delineating metabolic tumor volume, evaluating the tumor metabolic load and as a reference for treatment response is better than MRI. The metabolic information of ^18^F-FDG PET/CT has improved the diagnostic evaluation of a number of human malignancies [28–31]. However, ^18^F-FDG is limited by high uptake in normal brain, that interfere with the identification of glioma and normal brain. Two major advantages of ^18^F-labeled AAs for glioma imaging are their relatively low uptake and retention in normal brain and ability to visualize the entire glioma volume, compared with ^18^F-FDG PET/CT [7, 27, 32]. ^18^F-labeled AAs that useful for glioma PET/CT imaging include ^18^F-FDOPA, ^18^F-OMFD, ^18^F-FET, ^18^F-FAMT, 2-FTyr, ^18^F-BPA, ^18^F-FSPG and ^18^F-FGln, the most commonly used are ^18^F-FDOPA and ^18^F-FET. Both visual and semiquantitative indices of ^18^F-FDOPA PET detected glioblastoma recurrence with high accuracy and were predictive for PFS (progression-free survival) [33]. There was a study suggests that ^18^F-FET PET/CT adds valuable diagnostic information in brainstem and spinal cord glioma, particularly when the diagnostic information derived from MRI is equivocal [34]. A systematic review and meta-analysis indicates that ^18^F-FET PET/CT demonstrated excellent performance for diagnosing primary brain tumors [35]. ^18^F-FET may also be used for distinguishing recurrent brain metastasis from radiation necrosis after radiation therapy [36], but additional data are needed in this field. ^18^F-FDOPA uptake on PET was associated with IDH (isocitrate dehydrogenase) mutation in newly diagnosed gliomas [37]. ^18^F-FDOPA PET/CT and fused ^18^F-FDOPA PET/MRI are also used for detecting striatal involvement in children with gliomas [38]. ^18^F-BPA (boron phenylalanine) is used for the tumor/ normal tissue ratio in boron neutron capture therapy of gliomas and other head and neck cancers [39–41]. ^18^F-FSPG was a novel PET radiopharmaceutical which demonstrated high uptake in intracranial malignancies studies of both small animal and human [42]. ^18^F-FGln showed high uptake in gliomas but low background brain uptake, facilitating clear tumor delineation [43–46].

### Neuroendocrine tumors

Neuroendocrine tumors (NETs) are a class of heterogeneous tumors that originate from peptidergic neurons and neuroendocrine cells, arise in different anatomic locations and are associated with symptoms resulting from the hormones or vasoactive peptides. Neuroendocrine tumors include carcinoid tumors, pheochromocytomas and paragangliomas, pancreatic islet cell tumors, medullary thyroid cancer, neuroblastoma, and small cell lung cancer [7, 47]. These tumors have been known as tumors with amine precursor uptake and decarboxylation (APUD) [48, 49]. APUD tumor cells can uptake and decarboxylate amine precursor such as 5-hydroxy-L-tryptophan (5-HTP) and L-dihydroxyphenylalanine (L-DOPA) and pass through aromatic L-amino acid decarboxylase (AADC) to decarboxylate them to the corresponding 5-hydroxy-L-tryptamine and dopamine. ^18^F-FDOPA has proved a valuable tool for the assessment of neuroendocrine tumors. ^18^F-FDOPA PET/CT is highly sensitive in posttreatment evaluation of patients with pheochromocytomas and paragangliomas, and better than MRI and CT [50–52]. ^18^F-FDOPA is also suited for imaging gastroenteropancreatic neuroendocrine tumors and neuroblastoma [53–56]. ^18^F-FDOPA PET/CT detected more positive body regions and lesions of carcinoid tumors than the combination of CT and SRS [55, 57–61]. ^18^F-FDOPA may play a potentially useful role in medullary thyroid cancer PET imaging as a better or at least complementary model [55, 62, 63]. However, for pancreatic islet cell tumors, the ^18^F-FDOPA PET is not significant [55, 64–66].

### Prostate cancer

According to the WHO global tumor epidemiology statistics (GLOBOCAN 2008), prostate cancer in 2008 ranked second in global male malignancy incidence (second only to lung cancer), accounting for 14% of all men with cancer [67]. CT and MR imaging has limited accuracy to detect the primary tumor and regional lymph node metastases. Typically, prostate cancer has a lower ^18^F-FDG uptake rate. Recent studies with ^18^F-FACBC and ^18^F-FACPC have shown that these ^18^F-labeled AA tracers can accurately detect tumor and regional lymph node metastases with better specificity and sensitivity [7, 68, 69]. Because ^18^F-FACBC is slowly excrete into the bladder, the background radioactivity in the pelvic cavity is low, and the tumor and lymph node metastasis of primary and recurrent prostate cancer can be clearly visualized [70]. In addition, there were studies show that ^18^F-FACBC was superior in detecting prostate cancer recurrence in patients with recurrent prostate cancer compared with ^111^In-capromab or ^11^C-choline [71–74]. Since the half-life of ^11^C is only 20.3 minutes, the use of ^11^C-choline PET is limited in institute with on-site cyclotron. Furthermore, in the case of natural amino acids such as L-^11^C-methionine (MET), rapid metabolism usually produces radiolabeled metabolites, which can confuse kinetic analysis and reduce image quality [75, 76]. ^18^F-FACBC could be considered an alternative tracer superior to ^11^C-choline in the setting of patients with biochemical recurrence after radical prostatectomy [71, 77–80]. ^18^F-fluciclovine PET/CT is also used for distinguishing between prostate tumours and benign tissue and for assessment of tumour aggressiveness [81]. A study of ^18^F-FACBC PET/CT used in the planning of radiation therapy for prostate cancer patients has also been reported [82].

### Breast cancer

Recent studies [83–85] have shown that ^18^F-fluciclovine that is a leucine analog radioactive tracer can also be used for breast cancer PET/CT imaging. ^18^F-fluciclovine PET/CT visualizes malignant tumors including invasive lobular breast cancer (ILC) and invasive ductal breast cancer (IDC). In primary and metastatic breast cancers, ^18^F-fluciclovine uptake was significantly higher than benign breast lesions and normal breast tissue. Changes in ^18^F-fluciclovine avidity were strongly associated with a reduction in the percentage of tumor on pathology caused by treatment [3]. In addition to detecting and locating breast cancer, ^18^F-fluciclovine may provide a new tool for the exploration of amino acid transport and metabolism in breast cancer. ^18^F-fluciclovine also detected lymph nodes and bone metastases, but liver metastases were less effective due to the high physiological uptake of the tracer in liver parenchyma. The highest uptake of ^18^F-fluciclovine appears in Nottingham grade 3 cancers and triple-negative breast cancers, suggesting that ^18^F-fluciclovine may play a role in identifying more aggressive malignancies [83–85]. ^18^F-FASu may serve as a valuable target for the diagnosis and treatment monitoring of certain breast cancers and may provide more sensitive detection than ^18^F-FDG in certain tumors [86, 87]. ^18^F-FGln PET could be used to track cellular glutamine pool size of triple-negative breast cancers [88].

## CONCLUSIONS

^18^F-labeled amino acids have been developed for preclinical and clinical tumor PET/CT imaging. ^18^F-FDOPA and ^18^F-FET are well established for diagnosis, monitoring response to therapy, staging, and assessing for recurrence of gliomas. ^18^F-FDOPA has proved a valuable tool for the assessment of neuroendocrine tumors. It is highly sensitive in posttreatment evaluation of patients with pheochromocytomas and paragangliomas, and suited for imaging gastroenteropancreatic neuroendocrine tumors and neuroblastoma. Studies with ^18^F-FACBC and ^18^F-FACPC have shown that these ^18^F-labeled AA tracers can accurately detect tumor and regional lymph node metastases of prostate cancer with better specificity and sensitivity. ^18^F-fluciclovine used in breast cancer PET/CT imaging have been reported. ^18^F-fluciclovine PET/CT visualizes malignant tumors including invasive lobular breast cancer (ILC) and invasive ductal breast cancer (IDC). In primary and metastatic breast cancers, ^18^F-fluciclovine uptake was significantly higher than benign breast lesions and normal breast tissue. In the future, we have some innovative and interesting ^18^F-labeled amino acid anologues available, such as ^18^F-BAAs (boramino acids), which demonstrated distinctly high AA transporter-mediated tumor uptake and rapid clearance from normal organs and tissues [7]. However, the role of ^18^F-labeled amino acids PET will be limited to diagnostic imaging only. In the era of theranostic medicine, the peptide-receptor imaging and further peptide-receptor radionuclide therapy (PRRT) are emerging for somatostatin-related neuroendocrine tumors and prostate cancers, for example, DOTA-TOC and DOTATATE PET/CT [89–93].
